# Translation of curative therapy concepts with T cell and cytokine antibody combinations for type 1 diabetes reversal in the IDDM rat

**DOI:** 10.1007/s00109-020-01941-8

**Published:** 2020-06-30

**Authors:** Anne Jörns, Tanja Arndt, Shinichiro Yamada, Daichi Ishikawa, Toshiaki Yoshimoto, Taivankhuu Terbish, Dirk Wedekind, Peter H. van der Meide, Sigurd Lenzen

**Affiliations:** 1 Institute of Clinical Biochemistry, Hannover Medical School, Hannover, Germany; 2 Institute of Experimental Diabetes Research, Hannover Medical School, 30625 Hannover, Germany; 3 Institute of Laboratory Animal Science, Hannover Medical School, Hannover, Germany; 4 Cytokine Biology Unit, Central Laboratory Animal Institute, Utrecht University, Utrecht, The Netherlands

**Keywords:** Antibody combination therapy, Cytokines, LEW.1AR1-*iddm* rat, Pancreatic beta cells, Reversal, Type 1 diabetes mellitus

## Abstract

**Abstract:**

Proinflammatory cytokines released from the pancreatic islet immune cell infiltrate in type 1 diabetes (T1D) cause insulinopenia as a result of severe beta cell loss due to apoptosis. Diabetes prevention strategies targeting different cytokines with antibodies in combination with a T cell antibody, anti-TCR, have been assessed for therapy success in the LEW.1AR1-*iddm* (IDDM) rat, an animal model of human T1D. Immediately after diabetes manifestation, antibody combination therapies were initiated over 5 days with anti-TNF-α (tumour necrosis factor), anti-IL-1β (interleukin), or anti-IFN-γ (interferon) together with anti-TCR for the reversal of the diabetic metabolic state in the IDDM rat. Anti-TCR alone showed only a very limited therapy success with respect to a reduction of immune cell infiltration and beta cell mass regeneration. Anti-TCR combinations with anti-IL-1β or anti-IFN-γ were also not able to abolish the increased beta cell apoptosis rate and the activated immune cell infiltrate leading to a permanent beta cell loss. In contrast, all anti-TCR combinations with anti-TNF-α provided sustained therapy success over 60 to 360 days. The triple combination of anti-TCR with anti-TNF-α plus anti-IL-1β was most effective in regaining sustained normoglycaemia with an intact islet structure in a completely infiltration-free pancreas and with a normal beta cell mass. Besides the triple combination, the double antibody combination of anti-TCR with anti-TNF-α proved to be the most suited therapy for reversal of the T1D metabolic state due to effective beta cell regeneration in an infiltration free pancreas.

**Key messages:**

- Anti-TCR is a cornerstone in combination therapy for autoimmune diabetes reversal.
- The combination of anti-TCR with anti-TNF-α was most effective in reversing islet immune cell infiltration.
- Anti-TCR combined with anti-IL-1β was not effective in this respect.
- The combination of anti-TCR with anti-TNF-α showed a sustained effect over 1 year.

**Electronic supplementary material:**

The online version of this article (10.1007/s00109-020-01941-8) contains supplementary material, which is available to authorized users.

## Introduction

New immunomodulatory intervention therapies to counteract beta cell loss due to type 1 diabetes (T1D) development must target proinflammatory cytokines released from activated immune cells to counteract their beta cell toxic potential [1–4]. It is general consensus nowadays that therapy success requires immunomodulatory combination therapies with different antibodies [5–9]. To be successful, these therapies have to target in particular the two main proinflammatory cytokines, namely IL (interleukin)-1β and TNF (tumour necrosis factor)-α, in the pancreatic islet immune cell infiltrate [1–3]. Successful combination therapies require in addition the inclusion of a T cell–specific antibody, as a cornerstone antibody, against the TCR/CD3 (T cell receptor/cluster of differentiation) complex, as documented in the T1D situation both in humans [10–14] and rat models of autoimmune diabetes [15, 16].

In order to identify a therapeutic antibody combination with maximal curative potential suitable for translation to the patient with newly diagnosed T1D, we studied in the present investigation in the IDDM (LEW.1AR1-*iddm*) rat, a model of human T1D [17–19], a variety of combinations composed of two or three therapeutic antibodies. The analyses of different combinations made it possible to identify antibody combinations with optimal curative potential ideally suited for translation to the patient with T1D in the early phase after diabetes manifestation in order to reverse the diabetic metabolic state and insulin deficiency due to the reconstitution of an infiltration-free endocrine pancreas along with a full regeneration of the beta cell mass.

Since the pancreatic beta cell is so extremely vulnerable due to its low protection against stress [20] along with a limited beta cell regeneration potential [21–23], intensive efforts are required to establish effective combination therapies with a strong capacity for suppression of destructive islet immune cell infiltration and a high potential for beta cell regeneration. This need for combination therapies with high curative potential in T1D is not so evident in other autoimmune diseases such as rheumatoid arthritis and inflammatory bowel diseases [10, 13, 24–28], where the more modest therapy goal, namely a symptom-free remission of the disease, can be reached in many cases also with an anti-cytokine monotherapy, such as with anti-TNF-α [25, 26, 29, 30].

## Material and methods

### Animals

Congenic LEW.1AR1-*iddm* (IDDM) rats (for details, see http://www.mh-hannover.de/34926.html) were bred and maintained under standard conditions in the Central Animal Facility of Hannover Medical School with viral and genetic monitoring [16, 17, 19]. Experimental procedures were approved by the District Government of Hannover (LAVES, no 33-42502-05/958 & 509.6-42502-03/684 and 33.9-42502-04/16/2115) in accordance with the guidelines for the care and use of laboratory animals.

### Experimental groups

Different experimental groups with IDDM rats of both sexes were studied. Group 1 (*n* = 6) comprised healthy, normoglycaemic control rats; group 2 (*n* = 11) comprised diabetic rats treated for 5 consecutive days with anti-TCR alone (0.5 mg/kg b.wt. i.v.), an antibody directed against the *α/β* chains of the TCR/CD3 complex (R73, Serotec, Munich, Germany) [31]. Group 3 (*n* = 10) comprised diabetic rats treated for 5 consecutive days with anti-TCR with a rat-specific anti-IFN-γ (DB1, 0.1 mg/kg b.wt. i.v.) [32, 33]. Group 4 (*n* = 10) comprised diabetic rats treated for 5 consecutive days with anti-TCR and a rat-specific anti-IL-β (0.1 mg/kg b.wt. i.v., a custom-made monoclonal antibody with the epitope of silk6 clone [Eurogentec, Liege, Belgium]) [34]. Group 5 (*n* = 5) comprised diabetic rats treated with anti-TCR and anti-TNF-α (5 mg/kg b.wt. i.v.; kindly provided by Janssen Research & Development, Spring House, PA, USA) with a follow-up of 60 days and in group 6 (*n* = 6) with the same combination with a follow-up of 360 days. Group 7 (*n* = 9) comprised diabetic rats treated for 5 consecutive days with the triple combination of anti-TCR, anti-TNF-α, and anti-IL-1β. Group 8 (*n* = 6) comprised non-treated acutely diabetic control rats. No adverse events were observed in any treatment group. All therapies were started within 1 day after diabetes onset at blood glucose concentrations > 7.5 mmol/l with a disease manifestation age of around 60 days (between 50 and 80 days). Animals in groups 1 and 8 received injections of 0.9% NaCl solution.

### Pancreatic tissue and serum processing

To identify the changes in islets, one pancreas biopsy was performed on the day of diabetes manifestation, immediately before the start of therapy, and another at the end of therapy as described previously [35]. The remaining pancreas comprising head and tail was collected for analyses 60 days and after anti-TCR combination with anti-TNF-α also 360 days (group 6) after the end of therapy including serum samples. Blood glucose concentrations were determined daily (Glucometer Elite®, Bayer, Leverkusen, Germany). Serum C-peptides were analysed with a rat-specific ELISA (Mercodia, Uppsala, Sweden) and serum cytokine protein concentrations with a multiplex immunoassay kit (Bio-Rad, Munich, Germany) [15, 16].

### Morphological analyses

Pancreatic sections were stained either with the avidin-biotin-complex or double immunofluorescence technique with primary antibodies for beta and immune cells [2, 16, 35]. The antibodies recognised epitopes other than those targeted by the therapeutic antibodies summarized in Supplementary Table S1. A minimum of 1000 beta cells were counted in the proliferation and apoptosis analyses. Twenty to 25 islets in pancreas biopsies and 30–50 islets in the organ were studied during treatment in each animal [15, 34, 35]. The beta cell mass, stained by insulin and GLUT2 on serial sections, was calculated as described before [15, 16]. Analyses were performed using an Olympus microscope BX61 and for scanning BX61VS (Olympus, Hamburg, Germany) [15, 16, 35].

### *In situ* reverse transcriptase-polymerase chain reaction

Gene expression changes in islets after the therapy-free interval were identified by *in situ* reverse transcriptase-polymerase chain reaction (*in situ* RT-PCR) analysis of pancreatic sections from all groups as described [15, 16, 35]. The used primer sequences are provided in Table S2.

### Statistical analyses

Results are presented as mean values ± SEM. Comparisons between the different therapy groups and the healthy or diabetic controls were analysed with ANOVA followed by Dunnett’s test or Bonferroni’s test for multiple comparisons with the Prism 5 program (Graphpad Inc, San Diego, CA, USA). Significance was accepted at *p* < 0.05.

## Results

### Metabolic effects after anti-TCR combination therapies with cytokine antibodies

**Fig. 1:**
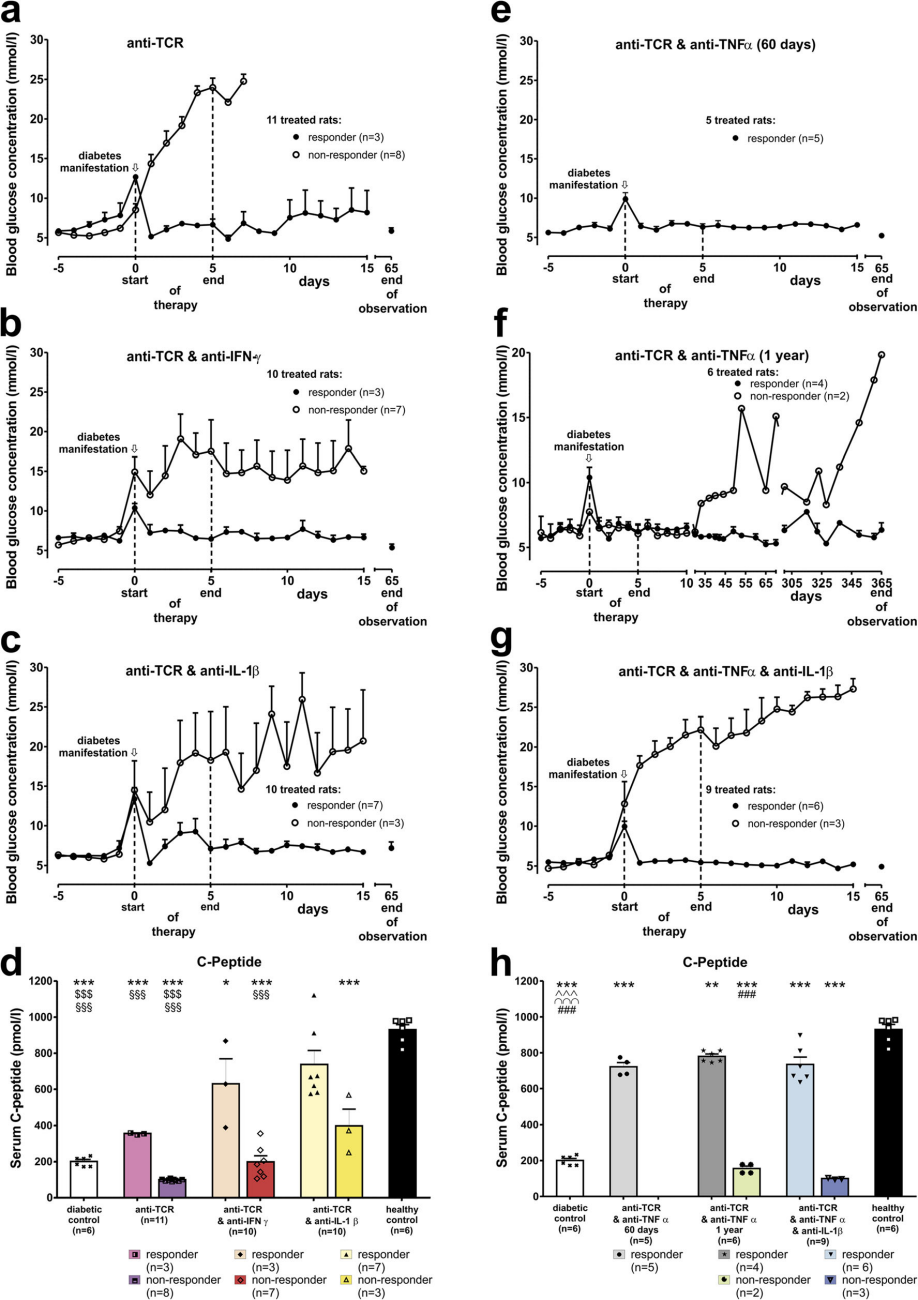
Effects of **a** anti-TCR alone and anti-TCR combination therapies, **b** with anti-IFN-γ, or **c** anti-IL-1β or **e** anti-TNF-α over 60 days and **f** over 360 days, and **g** in a triple combination with anti-TNF-α plus anti-IL-1β on the metabolic profile of IDDM rats after diabetes manifestation compared to diabetic control rats (first column in all graphs with columns) without treatment and to healthy control rats (last column in all graphs with columns). **a–c** Blood glucose concentration (mmol/l) changes are shown for the responding and non-responding rats in response **a** to anti-TCR or the different anti-TCR combination therapies **b** with anti-IFN-γ, **c** anti-IL-1β or **e** anti-TNF-α for 60 days, **f** anti-TNF-α over 1 year and **g** in a triple combination anti-TNF-α plus anti-IL-1β. The first dashed line at day 0 indicates the start of therapy and the second dashed line at day 5 indicates the end of therapy. **d**, **h** Serum C-peptide concentration changes (pmol/l) are shown for rats responding and non-responding **d** to anti-TCR alone or the different combination therapies of anti-TCR with anti-IFN-γ or anti-IL-1β or **h** for rats responding and non-responding to the different combination therapies of anti-TCR with anti-TNF for 60 days after the end of therapy or over 1 year, or in the triple combination. Data are mean values ± SEM. Comparison of the different experimental groups including diabetic and healthy controls by one way ANOVA followed by the Bonferroni test. Diabetic control or each experimental group ***p < 0.001, **p < 0.01, or *p < 0.05 to the healthy control, ^$$$^*p* < 0.001 to anti-TCR combination with anti-IFN-γ, ^§§§^*p* < 0.001 to anti-TCR combination with anti-IL-1β, ^^^^^*p* < 0.001 to anti-TCR combination with anti-TNF-α 60 days, ^∩∩∩^*p* < 0.001 to anti-TCR combination with anti-TNF-α 360 days, ^###^*p* < 0.001 to anti-TCR combination with anti-TNF-α plus anti-IL-1β

All therapies started within 1 day after diabetes manifestation (blood glucose > 7.5 mmol/l) in diabetic IDDM rats. Monotherapy with anti-TCR was given for 5 consecutive days (Fig. 1a). At most, transient instable glycaemia was achieved in very few of the treated diabetic rats (3/11) (Fig. 1a). Anti-TCR combined with either anti-IFN-γ, anti-IL-1β, or anti-TNF-α was administered for 5 consecutive days to the rats. With all three treatments, reductions of blood glucose values below 6 mmol/l were achieved in the presence of anti-TCR (Fig. 1b, c, e). However, a return to normoglycaemia from initial blood glucose values in the diabetic range between 8 and 15 mmol/l 60 days after the end of therapy was achieved only in less than 1/3 of the animals (3/10) treated with anti-IFN-γ (5.8 ± 0.2 mmol/l) (Fig. 1b) but in around 2/3 of the animals treated with anti-IL-1β (7/10) (5.6 ± 0.2 mmol/l) (Fig. 1c), and in all animals treated with anti-TNF-α (5/5) (5.2 ± 0.2 mmol/l) corresponding to healthy control animals (5.2 ± 0.2 mmol/l) (Fig. 1e).

In the successfully treated diabetic animals with the different anti-TCR combination therapies with anti-IFN-γ or anti-IL-1β, serum C-peptide concentrations doubled 60 days after the end of therapy compared with the values of the diabetic animals (200 ± 16 pmol/l) (first column) before the start of therapy or to the monotherapy with anti-TCR (356 ± 3 pmol/l) (Fig. 1d).

When animals were treated with anti-TCR and anti-TNF-α and kept either for 60 days or for a 1-year observation period (360 days after the end of therapy), therapy success could be maintained during this long observation period in 4 out of 6 rats (6.4 ± 0.5 mmol/l) (Fig. 1f) compared with a complete therapy success after the shorter period (5/5) (Fig. 1e).

Finally, a triple combination treatment for anti-IL-1β and anti-TNF-α with anti-TCR was tested. This was the most successful treatment combination with a mean blood glucose of 5.0 ± 0.2 mmol/l in 6 out of 9 rats at day 60 (Fig. 1g) being not significantly different from healthy control rats (last column).

In the successfully treated diabetic animals with the different anti-TCR combination therapies with anti-TNF-α, serum C-peptide concentrations were increased three- to fourfold 60 days after the end of therapy compared with the values of the rats in the diabetic control group (200 ± 16 pmol/l) (first column) before the start of therapy (Fig. 1h).

**Fig. 2:**
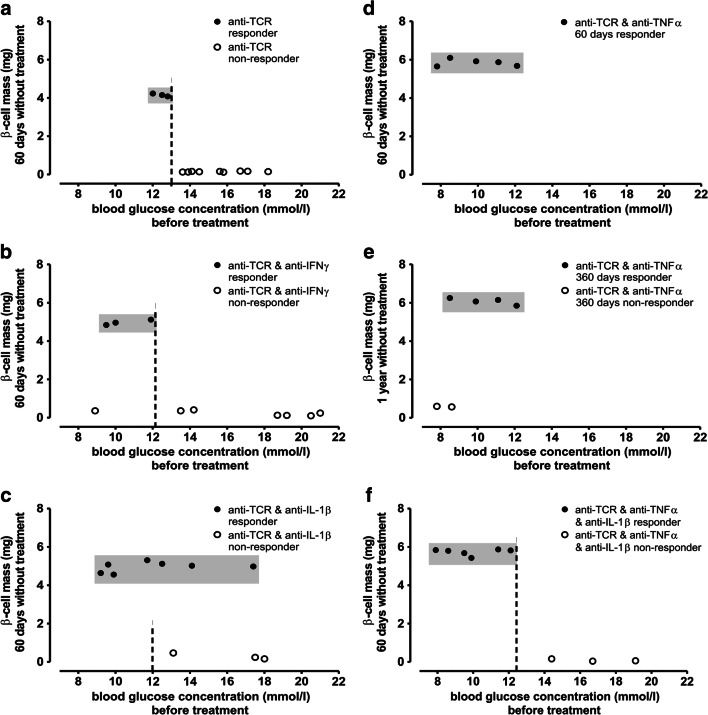
Relation between blood glucose concentration (mmol/l) at the start of therapy and beta cell mass (mg) **a–c**, **d**, **f** 60 days or **e** 1 year after the end of therapy in IDDM rats. **a** After anti-TCR alone and **b** after anti-TCR combination therapy with anti-IFN-γ, **c** with anti-IL-1β, **d** with anti-TNF-α all followed 60 days, **e** with anti-TNF-α followed for 1 year **f** in the triple combination with anti-TNF-α plus anti-IL-1β followed 60 days. The therapy success differed between the six analysed therapeutic groups either in the starting point of the blood glucose concentrations for the reversal to normoglycaemia or in the increase of beta cell mass, over 5 mg or around 6 mg representing the beta cell mass of healthy control animals

The different antibody therapies increased beta cell mass from initial values of around 2 mg in the diabetic control group before the start of therapy to values approaching 4 mg with the anti-TCR monotherapy (Fig. 2a) and those of the healthy control rats (around 6 mg) with distinct combination therapies (Fig. 2b–f). However, the efficiency of treatment differed between the different treatment groups reaching beta cell mass values above 5 mg at the end of the 60-day observation period (Fig. 2b–d, f). Interestingly, however, this therapy success could be achieved in the anti-TCR combination therapy with anti-IFN-γ typically only in animals with initial blood glucose concentration values below 12 mmol/l (Fig. 2b). In the combination therapy with anti-IL-1β, it was possible to regain beta cell mass values of around 5 mg even in half of the rats with starting blood glucose values above 12 mmol/l (Fig. 2c). Anti-TCR combination therapy with anti-TNF-α was most effective in terms of beta cell mass values in the range of 6 mg (Fig. 2d).

After a 1 year observation period in the animals treated with anti-TCR plus anti-TNF-α, the beta cell mass increase remained in the same range (Fig. 2e).

In the triple combination, all 6 animals with blood glucose values of up to 13 mmol/l showed a therapy success with a beta cell mass increase to around 6 mg (Fig. 2f).

### Quantification of the effects of anti-TCR combination therapies with cytokine antibodies

#### Changes in beta cell proliferation and apoptosis rates

**Fig. 3:**
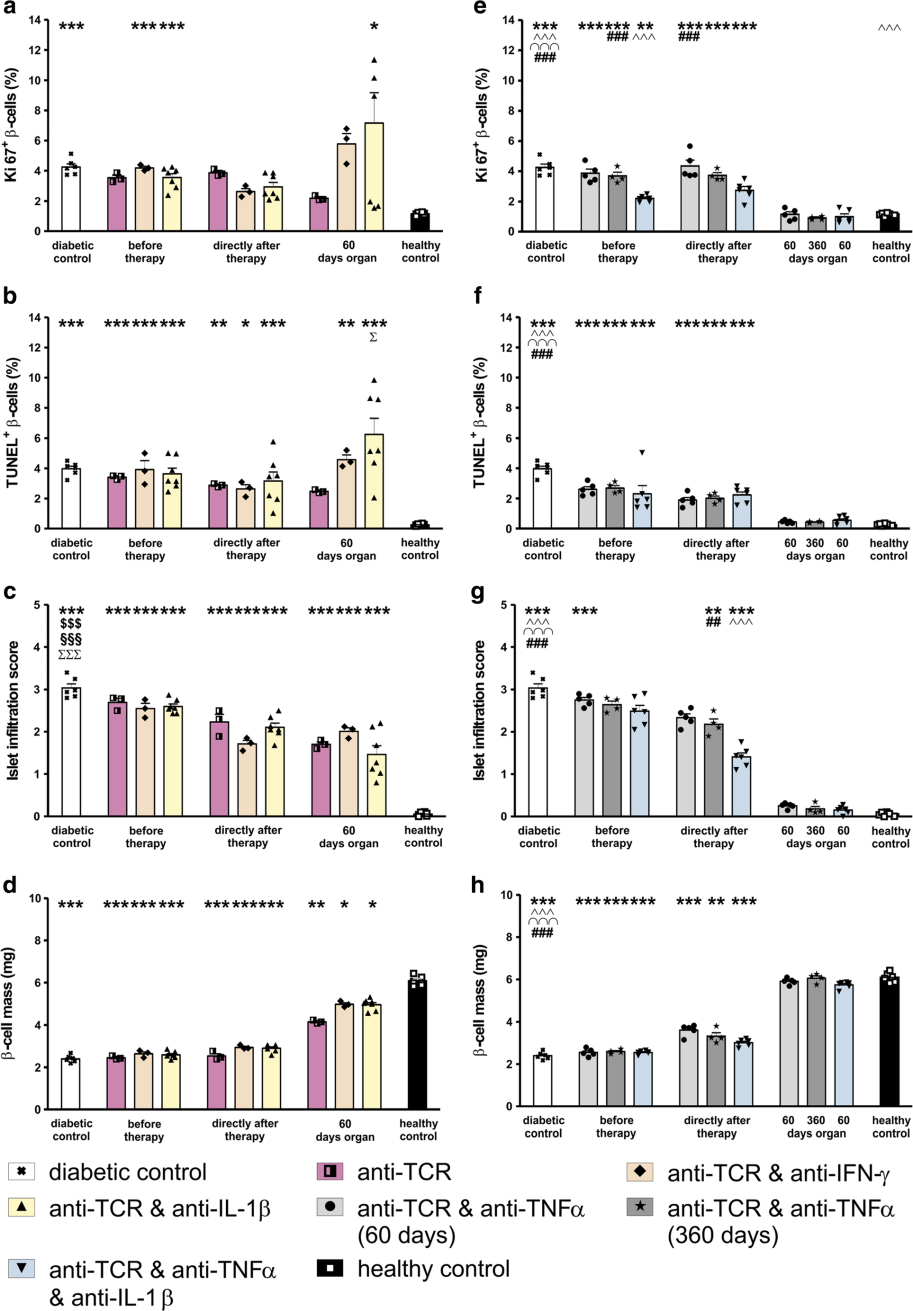
Morphometric analyses of beta cells and immune cells in IDDM rats after anti-TCR monotherapy and after successful anti-TCR combination therapy after diabetes manifestation with anti-IFN-γ, anti-IL-1β, and anti-TNF-α followed for 60 days after the end of therapy or with anti-TNF-α followed for 360 days, or combined with anti-IL-1β followed for 60 days after the end of therapy. Changes in the rate of **a**, **e** proliferation; **b**, **f** apoptosis; **c**, **g** islet infiltration score; and **d**, **h** pancreatic beta cell mass. Measurements were performed immediately before therapy, at the end of therapy, and 60 or 360 days after the end of therapy. Data are mean values ± SEM. Comparison of the different experimental groups including diabetic and healthy controls by one way ANOVA followed by the Bonferroni test. Diabetic control (first column in all graphs) or each experimental group ***p < 0.001, **p < 0.01, or *p < 0.05 to the healthy control (last column in all graphs). ^Σ^*p* < 0.05 to anti-TCR monotherapy, ^$$$^*p* < 0.001 to anti-TCR combination with anti-IFN-γ, ^§§§^*p* < 0.001 to anti-TCR combination with anti-IL-1β, ^^^^^*p* < 0.001 or ^^^^*p* < 0.01 to anti-TCR combination with anti-TNF-α 60 days, ^∩∩∩^*p* < 0.001 to anti-TCR combination with anti-TNF-α 360 days, ^###^*p* < 0.001or ^##^*p* < 0.01 to anti-TCR combination with anti-TNF-α plus anti-IL-1β

At the day of diabetes manifestation, immediately before the start of therapy, the diabetic rats that were responsive to the anti-TCR therapy alone or in combination with anti-IFN-γ or with anti-IL-1β showed a significant more than threefold increase of the proliferation rate analysed by Ki67 staining (Fig. 3a); the apoptosis rate increased more than 30-fold (Fig. 3b) compared with healthy controls. The proliferation rate in rats treated with anti-TCR and anti-TNF-α for 60 or 360 days or with the triple combination (Fig. 3e) showed also a threefold increase and the apoptosis rate a 20-fold increase (Fig. 3f). All these values before the start of therapy corresponded also to the values observed in diabetic control animals (Fig. 3a, b, e, f).

After the end of the monotherapy and combination therapies of anti-TCR with the different cytokine antibodies, the rates of proliferation (Fig. 3a, e) as well as of apoptosis (Fig. 3b, f) remained increased.

Even at 60 days after the end of therapy, in the responding rats with the combinations of anti-TCR plus anti-IFN-γ or anti-IL-1β, the proliferation rates (four to eightfold) as well as the apoptosis rates remained increased (20- till 30-fold) compared with those of healthy controls (Fig. 3a, b).

Only after the combination of anti-TCR with anti-TNF-α the proliferation rate (Fig. 3e) and apoptosis rate (Fig. 3f) returned to values in the range of the healthy control rats. The same was true for the proliferation and apoptosis rate after 1 year for this combination (Fig. 3e, f), documenting the stability of the therapeutic effect. An apoptosis rate in the normal range as an optimal result was achieved also with the triple antibody combination therapy (Fig. 3e, f).

#### Infiltration score

Before the start of therapy, the infiltration score of the islets was high with values of around 3 for all therapies (Fig. 3c, g). The insulitis score was marginally reduced immediately after the end of all combination therapies (Fig. 3c, g). At 60 days after the end of therapy, the infiltration score in the regenerated endocrine pancreases was only slightly reduced to values of around 2 for the anti-TCR monotherapy and the anti-TCR combination with anti-IFN-γ and to values of around 1 for the anti-TCR combination with anti-IL-1β (Fig. 3c). In all combination therapies of anti-TCR with anti-TNF-α including the combination plus anti-IL-1β, the infiltration score values were in the low range of the control values (0.1–0.3) independent from the observation period (60 or 360 days) (Fig. 3g).

#### Beta cell mass

After diabetes manifestation and before therapy, the beta cell mass was reduced in all diabetic rats to around 1/3 of the value in normoglycaemic healthy controls (Fig. 3d, h). Without treatment, these residual beta cells are lost within 1 week. Immediately after the end of anti-TCR alone or the anti-TCR combination therapies with the different cytokine antibodies, the respective beta cell mass remained low (Fig. 3d, h). The exception was the combination of anti-TCR with TNF-α, which showed already a clear beta cell mass increase immediately after the end of therapy (Fig. 3h).

Sixty days after the end of the different combination therapies, the beta cell mass had attained values approaching the normal range with the highest beta cell mass values comparable with healthy controls achieved in the combination therapies of anti-TCR with anti-TNF-α including the triple combination (Fig. 3h). In contrast, in the few partially successful combination therapies with anti-IL-1β and in particular with anti-IFN-γ beta cell mass increase was rather limited (Fig. 3d).

### Islet immune cell infiltration pattern after anti-TCR combination therapies with cytokine antibodies

**Fig. 4:**
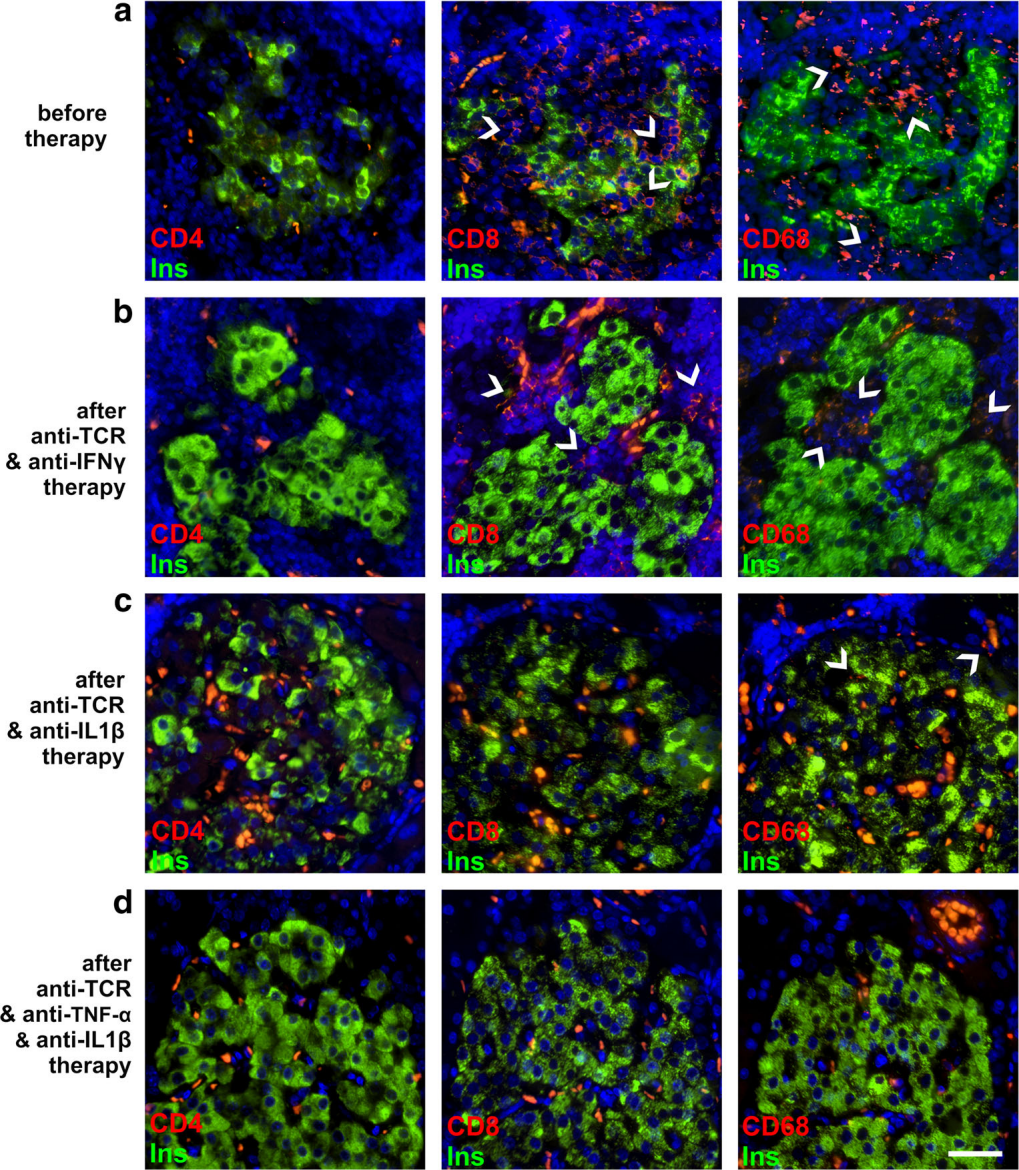
Immune cell infiltration in pancreatic islets of IDDM rats after successful anti-TCR combination therapy with anti-IFN-γ, anti-IL-1β, or in the triple combination after diabetes manifestation and after the treatment-free period of 60 days after the end of therapy. **a–d** Beta cells (*green*) and immune cells (*red*) were examined in islets from animals successfully treated with the anti-TCR combination with **b** anti-IFN-γ, **c** anti-IL-1β, or **d** in the triple fashion anti-TCR combined together with anti-TNF-α plus anti-IL-1β after diabetes manifestation and compared with **a** the untreated diabetic situation. Islets were immunostained for insulin (*green*) and CD4^+^ T cells (*red*), CD8^+^ T cells (*red*), and CD68 macrophages (*red*) and counterstained with DAPI (*blue*). Erythrocytes were identified by *yellow* to *orange* colour through auto-fluorescence in the red and green channels. The quantification of these immune cells revealed for the healthy control only macrophages with the absolute number of 2 immune cells/islet, whereas the diabetic control showed 42 immune cells/islet with 4% CD4^+^ T cells, 45% CD8^+^ T cells, and 51% CD68 macrophages; anti-TCR combination with anti-IFN-γ reduced the immune cells to 16/islet with 5% CD4^+^ T cells, 43% CD8^+^ T cells and 53% CD68 macrophages. After anti-TCR therapy with anti-IL-1β the absolute number of immune cells was reduced to 8/islet with a predominance of 85 % macrophages. After anti-TCR combination with anti-TNF-α alone (60 and 360 days) or in the triple fashion with anti-IL-1β, only macrophages were present in the same amount as in the islets of healthy controls. Scale bar represents 25 μm

After diabetes manifestation, the islet infiltrate was composed of a high number of both immune cell types, CD8^+^ T cells and CD68 macrophages, and to a lesser extent of around 10 % CD4^+^ T cells as well as 10 % of NK-cells and B cells in the acutely diabetic rats (Fig. 4a).

At 60 days after the end of the different anti-TCR combination therapies with anti-IFN-γ or anti-IL-1β, the responding rats showed still a significant residual immune cell infiltration in the islets (Fig. 4b, c). The same was true for the monotherapy with anti-TCR (not shown). After the triple anti-TCR combination therapy, however, no immune cell infiltration whatsoever was still present in the islets representing the situation in control islets (Fig. 4d).

### Changes of cytokines in pancreatic islets and serum after anti-TCR combination therapies with cytokine antibodies

#### Gene expression

Compared to non-infiltrated islets from normoglycaemic control rats, high levels of gene expression of the proinflammatory cytokines, *Ifng*, *Il1b*, *Tnf*, and *Il2* as well as the anti-inflammatory cytokines *Il4* and *Il10* were present in the infiltrating immune cells in the islets of diabetic rats (Table 1). Sixty days after the end of anti-TCR alone or of the anti-TCR combination therapy with anti-IFN-γ a marginal decrease and with anti-IL-1β, a moderate decrease was observed in the gene expression of proinflammatory cytokines in the immune cells, while the expression of anti-inflammatory cytokines was not significantly affected (Table 1). In contrast, the different combinations of anti-TCR with anti-TNF-α strongly reduced both the pro- and anti-inflammatory cytokine gene expression (Table 1).

**Table 1:** Cytokine and cell cycle marker gene expression by *in situ* RT-PCR in pancreatic islets of different therapies with anti-TCR alone or in combination with anti-IFN-γ, anti-IL-1β, or anti-TNF-α in double or triple fashion 60 days after the end of therapy or in combination with anti-TNF-α followed for 1 year compared with healthy and diabetic controls in IDDM rats

Gene	Diabetic control	Anti-TCR	Anti-TCR and anti-IFN-γ	Anti-TCR and anti-IL-1β	Anti-TCR and anti-TNF-α (60 days)	Anti-TCR and anti-TNF-α (360 days)	Anti-TCR, anti-IL-1β, and anti-TNF-α	Healthy control
IFN-γ	2.3 ± 0.1	1.7 ± 0.2	1.1 ± 0.1	0.8 ± 0.1	0.6 ± 0.1**	0.0 ± 0.0**	0.0 ± 0.0**	0.0 ± 0.0
IL-1β	21.8 ± 0.6	14.2 ± 0.3	10.5 ± 0.5*	2.4 ± 0.2**	4.7 ± 0.3**	3.8 ± 0.1**	0.6 ± 0.1**	0.1 ± 0.1
TNF-α	18.8 ± 0.8	12.6 ± 0.4	13.5 ± 0.4	4.8 ± 0.3**	0.7 ± 0.1**	0.0 ± 0.0**	0.0 ± 0.0**	0.0 ± 0.0
IL-2	2.5 ± 0.2	1.4 ± 0.1*	2.2 ± 0.2	0.5 ± 0.0**	0.1 ± 0.0**	0.1 ± 0.0**	0.2 ± 0.0**	0.0 ± 0.0
IL-4	5.5 ± 0.2	3.4 ± 0.2	5.3 ± 0.1	4.5 ± 0.2	0.7 ± 0.1**	0.6 ± 0.0**	0.3 ± 0.1**	0.0 ± 0.0
IL-10	3.2 ± 0.2	1.0 ± 0.2*	1.7 ± 0.3	4.7 ± 0.3	1.8 ± 0.2*	1.5 ± 0.1*	0.7 ± 0.0**	0.0 ± 0.0
Casp3	1.7 ± 0.0	1.7 ± 0.2	2.2 ± 0.1	1.3 ± 0.1	0.3 ± 0.0**	0.1 ± 0.0**	0.3 ± 0.0**	0.2 ± 0.0
PCNA	3.5 ± 0.2	2.5 ± 0.1	1.5 ± 0.1	2.8 ± 0.2	1.6 ± 0.1*	1.5 ± 0.1*	2.1 ± 0.1	1.1 ± 0.1

Gene expression for the pro- and anti-inflammatory cytokines IFN-γ (*Ifng*), IL-1β (*Il1b)*, TNF-α (*Tnf*), IL-2 (*Il2*), IL-4 (*Il4*), and IL-10 (*Il10*) as well as for caspase 3 (*Cpp32*), proliferating cell nuclear antigen (*PCNA*) in the islets. The quantitative analysis of the expression frequency in the immune cells was performed in the pancreases of the successfully treated rats in all groups (a total of 30 to 50 islets in each group) and calculated as a percentage of the positive mRNA transcript staining of the immune cell infiltration area for the cytokines and the islet area for caspase 3, and PCNA. The values were expressed as mean values ± SEM. *p < 0.05 and **p < 0.01 for all successful therapies versus the diabetic controls by ANOVA followed by Dunnett’s test. The number of animals as given in Fig. 1

In the triple combination therapy mode, pro- and anti-inflammatory cytokine gene expression was virtually completely abolished close to those values in islets of healthy controls (Table 1).

#### Protein expression

**Fig. 5:**
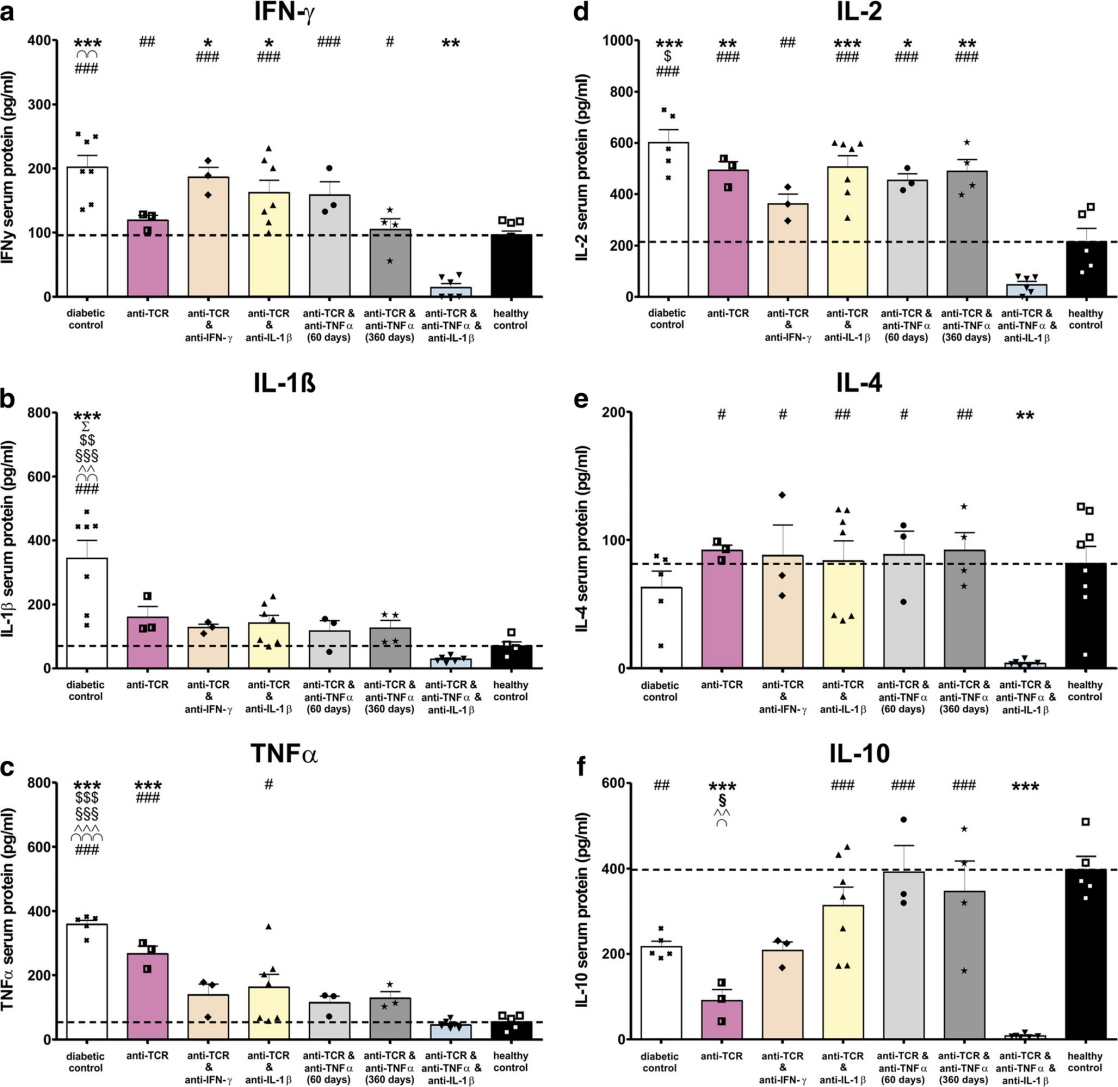
Cytokine pattern in serum of IDDM rats after anti-TCR alone and after successful anti-TCR combination therapies with anti-IFN-γ, anti-IL-1β, and anti-TNF-α all followed for 60 days and with anti-TNF-α also for 1 year or in triple combination followed for 60 days after diabetes manifestation. Changes in protein concentrations of cytokines measured by the multiplex analysis were examined **a** IFN-γ, **b** IL-1β, **c** TNF-α, **d** IL-2, **e** IL-4, and **f** IL-10. Results after anti-TCR alone and anti-TCR combination therapy with anti-IFN-γ, with anti-IL-1β, with anti-TNF-α for 60 days or 1 year, or triple fashion were compared with those from healthy control rats (last column) and diabetic control rats without treatment (first column). Cytokine protein concentrations (pg/ml) are expressed as mean values ± SEM; **a**–**f** the dotted lines show changes in the proinflammatory and anti-inflammatory cytokines compared with the normoglycaemic control. Comparison of the different experimental groups including diabetic and healthy controls by one way ANOVA followed by the Bonferroni test. Diabetic control (first column in all graphs) or each experimental group ***p < 0.001, **p < 0.01, or *p < 0.05 versus healthy controls (last column in all graphs), ^Σ^*p* < 0.05 to anti-TCR monotherapy, ^$$$^*p* < 0.001, ^$$^*p* < 0.01, or ^$^*p* < 0.05 to anti-TCR combination with anti-IFN-γ, ^§§§^*p* < 0.001 or ^§^*p* < 0.05 to anti-TCR combination with anti-IL-1β, ^^^^^*p* < 0.001 or ^^^^*p* < 0.01 to anti-TCR combination with anti-TNF-α 60 days, ^∩∩∩^*p* < 0.001, ^∩∩^*p* < 0.01 or ^∩^*p* < 0.05 to anti-TCR combination with anti-TNF-α 360 days, ^###^*p* < 0.001, ^##^*p* < 0.01, or ^#^*p* < 0.05 to anti-TCR combination with anti-TNF-α plus anti-IL-1β

Sixty days after the end of combination therapies with all cytokine antibodies, the serum protein concentrations of the proinflammatory cytokines were significantly reduced in particular in the combinations with anti-IL-1β and anti-TNF-α, when compared with diabetic control animals (Fig. 5a–c). After the triple combination therapy, the serum protein concentrations of the three proinflammatory cytokines were low or even lower than those in healthy control animals (Fig. 5a–c).

The high level of immune cell activating cytokine IL-2 decreased significantly in animals treated with the triple combination (Fig. 5d). The anti-inflammatory cytokines IL-4 and IL-10 which decreased in the diabetic control rats increased in animals treated with anti-TCR in combination with anti-IL-1β and anti-TNF-α (Fig. 5e, f).

The results document on the gene and protein level of pancreatic islets and serum cytokines a clear superiority of anti-TCR combinations with anti-TNF-α in all parameters over those with anti-IFN-γ or anti-IL-1β and with a further therapy optimization in the case of the triple antibody combination.

## Discussion

Successful therapy of T1D is dependent upon the administration of a CD3/TCR antibody to reverse the activation of the T cells in the immune cell infiltrate in the endocrine pancreas [12, 14, 24]. In addition, proinflammatory cytokines produced by different infiltrating immune cells play a crucial role in the pathogenesis of this autoimmune disease. In concert, the toxic action of the T cells and the proinflammatory cytokines mediate beta cell destruction in the islets of Langerhans, thereby causing an insulinopenia which results in the development of diabetic hyperglycaemia [3, 36–38]. Targeting single components of the autoimmune disease process did not yield sustained therapy success [5, 39]. It is therefore consensus in the scientific community that combination therapies are required to target the different components of the disease process and thereby reverse diabetic hyperglycaemia through suppression of the autoimmune-mediated beta cell destructive process and the subsequent regeneration of the insulin-producing cells [6–9].

The aim of this study was to device combination therapies with maximal curative potential in the IDDM rat, an animal model of autoimmune diabetes, which mirrors the human T1D situation optimally [2, 17, 19, 40]. The model is thus very much suited for the transferal of curative combination therapy concepts to patients with T1D in the early phase of the disease immediately after the manifestation of diabetic hyperglycaemia.

The results show that the combined administration of an antibody against TCR/CD3 and an antibody against TNF-α is the minimum requirement for achieving a long-term therapy success, as documented by a sustained suppression of autoimmunity in connection with the abolishment of the beta cell toxic effect of the proinflammatory cytokine TNF-α. Only this combination returned beta cell proliferation and apoptosis rates into the low range of healthy animals. The combination of anti-TCR with anti-IL-1β did not provide this sustained effect and a combination of anti-TCR with anti-IFN-γ was even more ineffective in the same way as the monotherapy with anti-TCR or anti-CD3 [13, 15, 41], thereby limiting the recovery of the beta cell mass. This explains also why the scientists of the Trial Network call this monotherapy a “disease-modifying therapy” [42] without curative potential. Even when these therapies showed a certain reduction of hyperglycaemia, only the combination of anti-TCR with anti-TNF-α provided this desired sustained curative effect, documented by a regain of an infiltration-free pancreas with sufficient beta cell mass along with a sustained fully normalized blood glucose profile.

The message from the present studies is therefore that a reduction of hyperglycaemia should not be taken as the sole indicator for therapy success, as it is often done in clinical studies, since this is not a valid parameter for proving curative therapy success.

Interestingly, the triple combination turned out to be the most effective therapeutic antibody combination yielding an optimal result with regenerated beta cells in the islets fully resembling in their structure healthy non-immune cell-infiltrated control islets. It was also the only therapy scheme that reduced all serum cytokines to levels below those of healthy control animals.

The key message from these studies with different combinations of antibodies is that for maximal therapy success, it is crucial to target the activated T cells with anti-TCR plus a targeting of the proinflammatory cytokines TNF-α and IL-1β with specific antibodies. Remarkable is in this context that this optimal therapy success was achieved with low doses of each of the antibodies in the combination, thereby minimizing the risk of serious side effects of this neutralizing immunomodulatory antibody therapy [24, 43]. Importantly, all these antibodies are also available as humanised antibodies and established in the therapies of a number of autoimmune diseases besides T1D such as rheumatoid arthritis and inflammatory bowel diseases [10, 13, 24–28]. Interestingly, antibodies against IFN-γ are also not established in the therapy of any other autoimmune disease [9, 44].

This combination therapy of anti-TCR with anti-TNF-α is a viable option especially in the LADA (latent autoimmune diabetes in adults) form of autoimmune diabetes in view of the slower disease progression both in LADA patients and in a rat model of human LADA than in patients and in the IDDM rat model with early manifesting T1D [36, 40, 45–48].

On the other hand, the triple combination as the most quickly acting and most effective combination therapy form should be the preferred treatment in all cases where the beta cell mass is already strongly reduced (< 30–40% of normal) and thus at the limit where it is still possible to initiate successfully a regeneration process of the residual beta cells strong enough for a regain of a functional beta cell mass required for restitution of normoglycaemia. This is likely to be the case when patients develop the disease at an early age. As it is the only combination which abolishes both the expression of TNF-α and IL-1β thereby maximally suppressing the beta cell destructive potential of the proinflammatory cytokines, it is optimally suited not only to prevent progression of beta cell loss but also the regain of a normal beta cell mass. This is a very urgent need in particular in children manifesting T1D, in whom beta cell loss is particularly quickly progressing. On the other hand, even the most effective therapy scheme did not yield the desired curative therapy results, when the residual beta cell mass was reduced by more than 70%, as documented in the present study in the rats that had blood glucose concentrations at therapy start in the range between 15 and 20 mmol/l. This documents the need for early therapy start also in T1D.

## Electronic supplementary material

ESM 1 (DOCX 21 kb)

## Data Availability

The datasets generated during and/or analysed during the current study are available from the author A.J. upon reasonable questions.

## Abbreviations

anti-TCR antibody: Anti–T cell receptor antibody
Casp3 (Cpp32): Caspase 3
IDDM rat: LEW.1AR1-*iddm* rat
IFN-γ: Interferon-gamma
IL-1β: Interleukin-1beta
TCR/CD3 complex: T cell receptor/cluster of differentiation complex
T1D: Type 1 diabetes
TNF-α: Tumour necrosis factor-alpha
