# Correction to: Translation of curative therapy concepts with T cell and cytokine antibody combinations for type 1 diabetes reversal in the IDDM rat

**DOI:** 10.1007/s00109-021-02151-6

**Published:** 2021-11-01

**Authors:** Anne Jörns, Tanja Arndt, Shinichiro Yamada, Daichi Ishikawa, Toshiaki Yoshimoto, Taivankhuu Terbish, Dirk Wedekind, Peter H. van der Meide, Sigurd Lenzen

**Affiliations:** 1 Institute of Clinical Biochemistry, Hannover Medical School, Hannover, Germany; 2 Institute of Experimental Diabetes Research, Hannover Medical School, 30625 Hannover, Germany; 3 Institute of Laboratory Animal Science, Hannover Medical School, Hannover, Germany; 4 Cytokine Biology Unit, Central Laboratory Animal Institute, UtrechtUniversity, Utrecht, The Netherlands

## Correction to: Journal of Molecular Medicine (2020) 98:1125–1137 https://doi.org/10.1007/s00109-020–01941-8

The article Translation of curative therapy concepts with T cell and cytokine antibody combinations for type 1 diabetes reversal in the IDDM rat by Anne Jörns, Tanja Arndt, Shinichiro Yamada, Daichi Ishikawa, Toshiaki Yoshimoto, Taivankhuu Terbish, Dirk Wedekind, Peter H. van der Meide and Sigurd Lenzen, was originally published online on 30 June 2020 without open access. With the author(s)’ decision to opt for Open Choice the copyright of the article changed on 30 September 2021 to © The Author(s) 2021 and the article is forthwith distributed under a Creative Commons Attribution 4.0 International License, which permits use, sharing, adaptation, distribution and reproduction in any medium or format, as long as you give appropriate credit to the original author(s) and the source, provide a link to the Creative Commons licence, and indicate if changes were made. The images or other third party material in this article are included in the article’s Creative Commons licence, unless indicated otherwise in a credit line to the material. If material is not included in the article’s Creative Commons licence and your intended use is not permitted by statutory regulation or exceeds the permitted use, you will need to obtain permission directly from the copyright holder. To view a copy of this licence, visit http://creativecommons.org/licenses/by/4.0.

The Original article has been corrected.

