# Supplementary material for: Translation of curative therapy concepts with T cell and cytokine antibody combinations for type 1 diabetes reversal in the IDDM rat
Source: J Mol Med (Berl). 2020 Jun 30;98(8):1125–37. doi: 10.1007/s00109-020-01941-8 (PMC8550584; doi:10.1007/s00109-020-01941-8)
Supplement: Supplementary file 1 — (DOCX 21 kb) [file 109_2020_1941_MOESM1_ESM.docx]

**Translation of curative therapy concepts with T cell and cytokine antibody combinations for type 1 diabetes reversal in the IDDM rat**

Anne Jörns^,^ Tanja Arndt, Shinichiro Yamada, Daichi Ishikawa, Toshiaki Yoshimoto, Taivankhuu Terbish, Dirk Wedekind, Peter H. van der Meide, Sigurd Lenzen

**Electronic Supplementary Material**

**Supplementary Table S1**

**Antibodies and their dilutions used for immunohistochemistry**

| **Peptide/protein**  **target** | **Clone** | **Manufacturer, cat. #,**  **and/or name of individual**  **providing the antibody** | **Species raised in**  **monoclonal or**  **polyclonal** | **Dilution**  **used** |
| --- | --- | --- | --- | --- |
| insulin |  | DAKO; A0564 | guinea pig; polyclonal | 100 |
| insulin (rat) | D3E7 | Bio-Rad, 5330-3369G | mouse; monoclonal | 600 |
| GLUT2  glucose transporter |  | Bio-Rad; 4670-1659 | rabbit; polyclonal | 1000 |
| CD8α | OX-8 | Bio-Rad; MCA48R | mouse; monoclonal | 100 |
| CD8ß | 341 | Bio-Rad; MCA938 | mouse; monoclonal | 100 |
| CD68 | ED1 | Bio-Rad; MCA341R | mouse; monoclonal | 100 |
| Ki 67 |  | Acris; AP00606PU-N | rabbit; polyclonal | 400 |
| CD3 | 1F4 | Bio-Rad; MCA772GA | mouse; monoclonal | 100 |
| CD4 | W3/25 | Bio-Rad; MCA55G | mouse; monoclonal | 100 |
| IL-1β |  | Bio-Rad; AAR15G | rabbit; polyclonal | 100 |
| IFN-γ | DB-1 | Bio-Rad; MCA1301 | mouse; monoclonal | 400 |
| TNFα |  | Bio-Rad; AAR33 | rabbit; polyclonal | 400 |
| IL-2 |  | R&D-Systems;  AF-502-NA | goat; polyclonal | 200 |
| IL-10 |  | Bio-Rad; AAR29 | rabbit; polyclonal | 100 |
| IL-17A (H-132) |  | Santa Cruz Biotechnology;  sc-7927 | rabbit; polyclonal | 100 |
| IL-17A |  | LSBio; LS-B13072 | rabbit; polyclonal | 200 |
| IL-6 |  | PeproTech; 500-P73G | goat; polyclonal | 200 |
| IL-4 |  | Bio-Rad; AAR16G | rabbit; polyclonal | 200 |

**Translation of curative therapy concepts with T cell and cytokine antibody combinations for type 1 diabetes reversal in the IDDM rat**

Anne Jörns^,^ Tanja Arndt, Shinichiro Yamada, Daichi Ishikawa, Toshiaki Yoshimoto, Taivankhuu Terbish, Dirk Wedekind, Peter H. van der Meide, Sigurd Lenzen

**Electronic Supplementary Material**

**Supplementary Table S2**

Sequences of primers used for *in situ* RT-PCR

| Gene | Accession Number | F (forward)  R (reverse) | Primer sequence |
| --- | --- | --- | --- |
| *Actb* | NM_031144 | F  R | 5’-ACAGCTGAGAGGGAAATCGT-3’  5’-CTGCTTGCTGATCCACATCT-3’ |
| *Il1b* | NM_031512 | F  R | 5’-GATGTTCCCATTAGACAGCTGCACTG-3’  5’-CTTTTCCATCTTCTTCTTTGGGTATTGT-3’ |
| *Ifng* | NM_138880 | F  R | 5’-GCTCTGCCTCATGGCCCTCTC-3’  5’-TGTTGCTGATGGCCTGGTTGTC-3’ |
| *Tnf* | X66539 | F  R | 5’‑CTACTGAACTTCGGGGTGATCGGTC-3’  5’-CTGGTATGAAGTGGCAAATCGGCT-3’ |
| *Il2* | NM_053836 | F  R | 5‘-TGGAGCAGCTGTTGCTGGAC-3‘  5‘-TGGCTCATCATCGAATTGGCACT-3‘ |
| *Il6* | NM_012589 | F  R | 5‘-CCAGTATATACCACTTCACAAGTCGGA-3‘  5‘-CAAGATGAGTTGGATGGTCTTGGTC-3‘ |
| *Il17* | NM_001106897 | F  R | 5‘-GGTACTCATCCCTACAAGTTCA-3‘  5‘-CTCTTCAGGACCAGGATCTCTT-3‘ |
| *Il4* | X16058 | F  R | 5’-TCTCAGCCCCCACCTTGCTG-3’  5’-TTGCGAAGCACCCTGGAAGC-3’ |
| *Il10* | NM_012854 | F  R | 5’-TGCACCCACTTCCCAGTCAGC-3’  5’-CACCTGCTCCACTGCCTTGC-3’ |

In control experiments performed without primers no specific staining was observed after the PCR reaction.
